# Phylogenetics, patterns of genetic variation and population dynamics of *Trypanosoma terrestris* support both coevolution and ecological host-fitting as processes driving trypanosome evolution

**DOI:** 10.1186/s13071-019-3726-y

**Published:** 2019-10-11

**Authors:** Sergio D. Pérez, Jared A. Grummer, Renata C. Fernandes-Santos, Caroline Testa José, Emília Patrícia Medici, Arlei Marcili

**Affiliations:** 1 Department of Preventive Veterinary Medicine and Animal Science, Faculty of Veterinary Medicine, University of São Paulo, São Paulo, Brazil; 2 Departamento de Biología, Facultad de Ciencias, Universidad del Tolima, Ibagué, Colombia; 3 Department of Zoology and Biodiversity Research Center, University of British Columbia, Vancouver, Canada; 4 Lowland Tapir Conservation Initiative (LTCI), Institute for Ecological Research (IPÊ), Campo Grande, Brazil; 5 Tapir Specialist Group (TSG), Species Survival Commission (SSC), International Union for Conservation of Nature (IUCN), Houston, USA; 6 Brazilian Institute for Conservation Medicine (TRÍADE), Campo Grande, Brazil; 7 Escola Superior de Conservação Ambiental e Sustentabilidade (ESCAS/IPÊ), Nazaré Paulista, Brazil; 8 Masters program in Medicine and Animal Welfare, Santo Amaro University, São Paulo, Brazil

**Keywords:** *Trypanosoma terrestris*, Ecological fitting, Coevolution, Population bottleneck, Microgeographic divergence, *Tapirus terrestris*, Perissodactyla

## Abstract

**Background:**

A considerable amount of evidence has favored ecological host-fitting, rather than coevolution, as the main mechanism responsible for trypanosome divergence. Nevertheless, beyond the study of human pathogenic trypanosomes, the genetic basis of host specificity among trypanosomes isolated from forest-inhabiting hosts remains largely unknown.

**Methods:**

To test possible scenarios on ecological host-fitting and coevolution, we combined a host capture recapture strategy with parasite genetic data and studied the genetic variation, population dynamics and phylogenetic relationships of *Trypanosoma terrestris*, a recently described trypanosome species isolated from lowland tapirs in the Brazilian Pantanal and Atlantic Forest biomes.

**Results:**

We made inferences of *T. terrestris* population structure at three possible sources of genetic variation: geography, tapir hosts and ‘putative’ vectors. We found evidence of a bottleneck affecting the contemporary patterns of parasite genetic structure, resulting in little genetic diversity and no evidence of genetic structure among hosts or biomes. Despite this, a strongly divergent haplotype was recorded at a microgeographical scale in the landscape of Nhecolândia in the Pantanal. However, although tapirs are promoting the dispersion of the parasites through the landscape, neither geographical barriers nor tapir hosts were involved in the isolation of this haplotype. Taken together, these findings suggest that either host-switching promoted by putative vectors or declining tapir population densities are influencing the current parasite population dynamics and genetic structure. Similarly, phylogenetic analyses revealed that *T. terrestris* is strongly linked to the evolutionary history of its perissodactyl hosts, suggesting a coevolving scenario between Perissodactyla and their trypanosomes. Additionally, *T. terrestris* and *T. grayi* are closely related, further indicating that host-switching is a common feature promoting trypanosome evolution.

**Conclusions:**

This study provides two lines of evidence, both micro- and macroevolutionary, suggesting that both host-switching by ecological fitting and coevolution are two important and non-mutually-exclusive processes driving the evolution of trypanosomes. In line with other parasite systems, our results support that even in the face of host specialization and coevolution, host-switching may be common and is an important determinant of parasite diversification.

## Background

A clear understanding of host–parasite relationships and the mechanisms by which parasites are adapted to their hosts is of paramount importance to public health, because emerging infectious diseases are frequently the result of host-switching, i.e. evolutionary change of host specificity of a parasite [1–3]. In contrast, emerging diseases can also be the result of the antagonistic coevolution between hosts and parasites, whereby hosts and pathogens display increased resistance and virulence in response to each other over time [4]. As a consequence, determining if parasite adaptation is due to host-switching or coevolution would provide valuable insights for the design and implementation of disease control programmes [5, 6]. However, distinguishing between coevolution and host-switching is often challenging because both can differ in their spatial and temporal scales [2, 7].

Parasites possess a great potential for evolutionary studies, having short generation times, complex life-cycles, and experiencing high fluctuations in population sizes, which in turn affect their population genetic structure [8, 9]. Thereby, combining micro- and macroevolutionary levels of genetic variation when studying parasite populations can shed light on which process is dominant, i.e. coevolution, host-switching, or a combination of both [10–12].

Strict cospeciation or long-term coevolution occurs when parasites and hosts speciate in synchrony [13]. Thus, host-switching may be rare, and both host–parasite phylogenies result in topological congruence and overall concordance in their divergence times [14]. Nonetheless, even though parasites indeed are highly specialized to their hosts, empirical evidence demonstrates host-switching rather than cospeciation as the dominant factor influencing the diversification of parasites [15]. Even in those cases where cospeciation occurs, frequently host-switching is involved [16]. This apparent ‘paradox’ (specialization *vs* host shifts) was solved in an empirical and theoretical framework by a process called ‘ecological fitting’, whereby host shifts can occur rapidly in ecological terms and the parasite should retain the capacity to use both the ancestral and novel host [17, 18]. This implies that the initial stages of colonization of new hosts do not require any evolutionary innovation, and the parasite co-opts an existing array of genetic traits to exploit and persist in such unfamiliar environments [17, 18]. Furthermore, host-switching by ecological fitting does not configure an evolutionary dead-end [5]. Further geographical range expansion of hosts and parasites and different biogeographical interactions (global episodes of climate change, allopatry, isolation, etc.) can foster novel selection regimes to parasite genotypes and eventually reach coevolutionary interactions and speciation [5, 7].

Trypanosomes (genus *Trypanosoma*) are a widespread and successful monophyletic group of kinetoplastid parasites that infect all vertebrate classes in all continents [19, 20]. They have complex life-cycles involving a vertebrate host and usually an arthropod or leech vector, in which several morphological and biochemical changes are needed for adaptation to the host and for transmission routes in different environments [21]. At least ten well-defined clades within the genus *Trypanosoma* are known (reviewed in [22]). Nevertheless, in spite of this high level of trypanosome diversity, researchers still debate whether coevolution or ecological host-fitting drives the evolution of trypanosomes [23]. One key example is *T. cruzi*, which has been assumed to coevolve tightly with triatomine vectors and mammal hosts, or if the parasite through its evolutionary radiation, has been able to colonize multiple hosts and niches by ecological fitting [24–27]. Although evidence favoring ecological fitting rather than cospeciation is increasing, most of the studies are macroevolutionary and examine broad phylogenetic patterns of divergence between trypanosomes and their hosts [28]. Despite notable efforts, population genetic studies are only restricted to medically important trypanosomes such as *T. cruzi* (e.g. [23, 26, 27]). For instance, at a macroevolutionary level, it has been established that success of ecological fitting in wild trypanosome species depends largely on the host range exploited by the invertebrate host [19, 28]. Hematophagous vectors such as leeches and arthropods are usually generalists and they do not infect a single vertebrate class, thereby during blood meals they facilitate the colonization and further adaptation of trypanosomes to several unrelated hosts [28]. Because most episodes of host-switching occur at fine ecological and microevolutionary scales [2], a complete understanding of such processes by means of population genetics and dynamics of wild trypanosomes is required.

To help fill these knowledge gaps and test alternative scenarios on ecological fitting and coevolution, we used new target isolates of *Trypanosoma terrestris*, a sylvatic trypanosome species first described in 2013 and retrieved from blood culture isolates of lowland tapirs (*Tapirus terrestris*; Perissodactyla: Tapiridae) in the Brazilian Atlantic Forest [29]. *Trypanosoma terrestris* is an ancient and divergent parasite that was proposed as a new clade and is thought to be specific to its vertebrate host as it apparently only grows in tapirs [29]. However, isolation of additional species of the *T. terrestris* clade from other perissodactyl hosts, pathogenicity, biological behavior in natural hosts, potential vectors, as well its evolutionary history remain unaddressed [29].

By using parasite genetic data and combining a micro- and macroevolutionary approach, as indicated previously [10–12], we test herein possible scenarios on ecological host-fitting and coevolution as follows. If *T. terrestris* exhibits a model compatible with coevolution, we expect a close association between parasite phylogeny and the evolutionary history of its perissodactyl hosts, including similar topology and divergence times. As occurs with specialist parasites [30], we predict a very limited parasite gene flow and therefore a high degree of genetic structure within and among tapir hosts, also restricted by biome or geographical barriers; and lastly, parasite population expansions or contractions constrained by the tapir host’s evolutionary history. Instead, if *T. terrestris* evolution is more compatible with a model of host-switching, we would observe phylogenetic patterns of parasite colonization to unrelated hosts (e.g. mammals and reptiles). Given that host-switching is a mechanism often exhibited by generalist parasites [31], the parasite genotypes should not be clustered by conspecific tapir hosts, implying high levels of gene flow and little or no genetic differentiation between parasites infecting different individual tapir hosts. Finally, as a consequence of vector-mediated host-switching, we would expect high fluctuations in parasite population sizes resulting in recurrent bottlenecks [32]. Our findings here support both coevolution and ecological host-fitting driving the evolution of *T. terrestris*. To the best of our knowledge, this is the first study highlighting the importance of both processes in trypanosomes, adding new lines of evidence and underlining the need to study sylvatic trypanosomes to understand the evolution and epidemiological features of human pathogenic trypanosomes.

## Methods

### Origin of *T. terrestris* isolates

*Trypanosoma terrestris* isolates were obtained from blood samples of wild lowland tapirs (*Tapirus terrestris*) from two different Brazilian biomes: Pantanal and Atlantic Forest. The Pantanal biome supports an abundance of wildlife and is the largest continuous freshwater wetland on earth (160,000 km^2^). Cattle ranching is the main economic activity in the region. This study was carried out on a private cattle ranch in the Nhecolândia subregion of the Pantanal, in the State of Mato Grosso do Sul, Brazil (19°20′S, 55°43′W). This private ranch is one of the study sites of the Lowland Tapir Conservation Initiative (LTCI), a pioneering long-term tapir research and conservation program carried out by the Institute for Ecological Research (IPE) in Brazil.

**Fig. 1:**
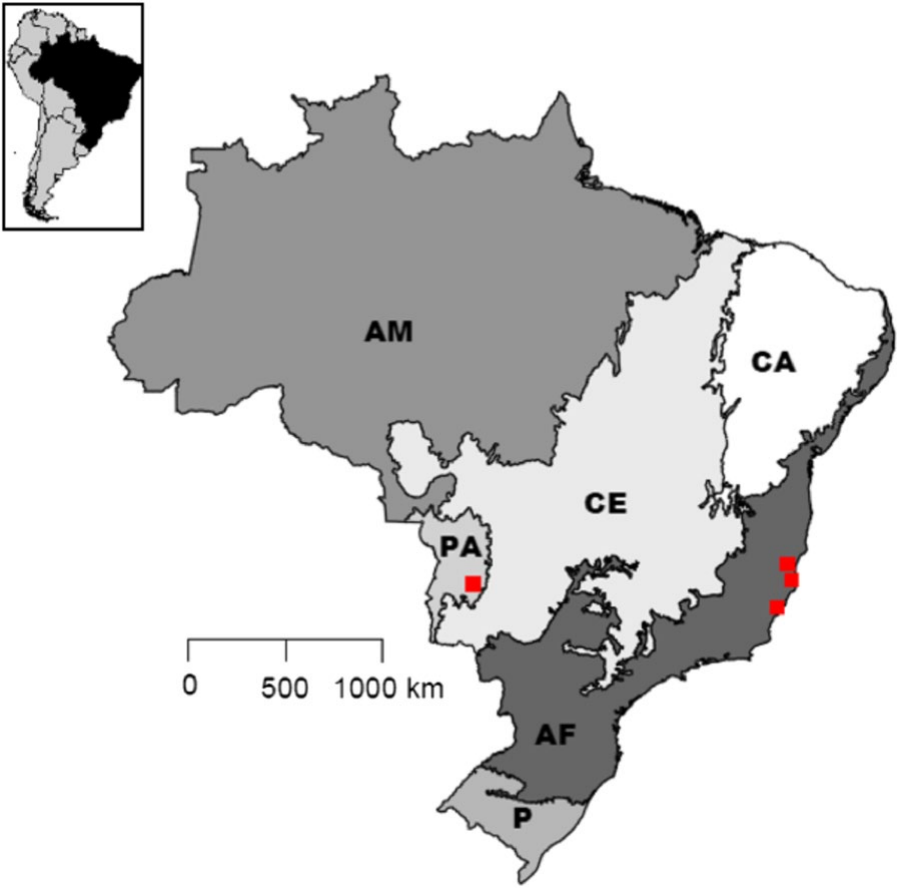
Positive isolates of *T. terrestris* according to their biome of origin. *Abbreviations*: AM, Amazonia; CE, Cerrado; PA, Pantanal; AF, Atlantic Forest; P, Pampa; CA, Caatinga

A total of 31 trypanosome isolates were included in this study: 28 new isolates from lowland tapirs in the Pantanal biome and three isolates from the original description of *Trypanosoma terrestris* in the Atlantic Forest biome. Information about parasite isolates and their tapir hosts and geographical locations is presented in Additional file 1: Table S1 and Fig. 1.

### Collection of blood samples, parasite isolation and culturing

At the Pantanal study site, the tapir capture methods included box traps and darting from a distance using anesthetic darts. Tapirs were immobilized using a combination of butorphanol, medetomidine and ketamine (atipamezole and naltrexone as antagonists). The handling included physical examination, anesthesia monitoring, microchip insertion, radio tagging, sexing and aging. To detect the presence of trypanosomes, blood samples were collected through venipuncture in rami of the saphenous or cephalic veins, using 5 ml syringes. Thirty-two blood samples from 24 wild lowland tapirs (12 males and 12 females) were collected in the Pantanal between September 2012 and December 2015. Ten drops of each blood sample were inoculated into vacutainer tubes containing a biphasic medium consisting of 10% sheep red blood cells as the solid phase (blood agar base), overlain by liquid LIT medium that was supplemented with 10% FBS. The resulting positive hemocultures were incubated at 28 °C and grown in LIT medium for DNA preparation, and the isolates (hereafter indistinctly called CBTs) were cryopreserved in liquid nitrogen in the Brazilian Trypanosomatid Collection (Coleção Brasileira de Tripanossomatídeos, CBT), in the Department of Preventive Veterinary Medicine and Animal Health, School of Veterinary Medicine, University of São Paulo, Brazil (Additional file 1: Table S1, Table 1).

**Table 1:** *Trypanosoma* isolates, host, geographical origin and sequences of *SSU* rDNA, ITS1 rDNA and *gGAPDH* gene used in the phylogenetic analyses

*Trypanosoma* spp.	CBT	Host species	Geographical origin		GenBank ID		
			Locality	State	*SSU* rDNA	*gGAPDH*	ITS1 rDNA
*T. terrestris*	CBT 46	*Tapirus terrestris*	Linhares	ES	KF586846	KF586843	**MK943702**
	CBT 60		Pinheiros	ES	KF586847	KF586440	**MK943703**
	CBT 61		Floriano Peixoto	ES	KF586848	KF586845	**MK943704**
	CBT 94		Nhecolândia	MS	**MK942351**	**MK944284**	**MK943705**
	CBT 97		Nhecolândia	MS	**MK942352**	**MK944285**	**MK943706**
	CBT 98		Nhecolândia	MS	**MK942353**	**MK944286**	**MK943707**
	CBT 101		Nhecolândia	MS	**MK942354**	**MK944287**	**MK943708**
	CBT 102		Nhecolândia	MS	**MK942355**	**MK944288**	**MK943709**
	CBT 103		Nhecolândia	MS	**MK942356**	**MK944289**	**MK943710**
	CBT 104		Nhecolândia	MS	**MK942357**	**MK944290**	**MK943711**
	CBT 109		Nhecolândia	MS	**MK942358**	**MK944291**	**MK943712**
	CBT 133		Nhecolândia	MS	**MK942359**	**MK944292**	**MK943713**
	CBT 134		Nhecolândia	MS	**MK942360**	**MK944293**	**MK943714**
	CBT 135		Nhecolândia	MS	**MK942361**	**MK944294**	**MK943715**
	CBT 140		Nhecolândia	MS	**MK942362**	**MK944295**	**MK943716**
	CBT 141		Nhecolândia	MS	**MK942363**	**MK944296**	**MK943717**
	CBT 142		Nhecolândia	MS	**MK942364**	**MK944297**	**MK943718**
	CBT 143		Nhecolândia	MS	**MK942365**	**MK944298**	**MK943719**
	CBT 164		Nhecolândia	MS	**MK942366**	**MK944299**	**MK943720**
	CBT 165		Nhecolândia	MS	**MK942367**	**MK944300**	**MK943721**
	CBT 167		Nhecolândia	MS	**MK942368**	**MK944301**	**MK943722**
	CBT 180		Nhecolândia	MS	**MK942369**	**MK944302**	**MK943723**
	CBT 181		Nhecolândia	MS	**MK942370**	**MK944303**	**MK943724**
	CBT 188		Nhecolândia	MS	**MK942371**	**MK944304**	**MK943725**
	CBT 189		Nhecolândia	MS	**MK942372**	**MK944305**	**MK943726**
	CBT 190		Nhecolândia	MS	**MK942373**	**MK944306**	**MK943727**
	CBT191		Nhecolândia	MS	**MK942374**	**MK944307**	**MK943728**
	CBT 192		Nhecolândia	MS	**MK942375**	**MK944308**	**MK943729**
	CBT 198		Nhecolândia	MS	**MK942376**	**MK944309**	**MK943730**
	CBT 199		Nhecolândia	MS	**MK942377**	**MK944310**	**MK943731**
	CBT 200		Nhecolândia	MS	**MK942378**	**MK944311**	**MK943732**
*T. vanstrieni*		*Dicerorhinus sumatrensis*		MY	GQ846158	GQ864159	
*T. grayi*		*Glossina palpalis*		AF	AJ620546	AJ620258	
*T. ralphi*		*Caiman yacare*		MS	EU596253	EU596257	
		*Caiman yacare*		MS	EU596254	EU596258	
		*Caiman crocodilus*		BR	KF546523	KF546514	
*T. terena*		*Caiman yacare*		MS	EU596252	EU596256	
*T. dionisii*		*Pipistrellus pipistrellus*		UK	AJ009151	AJ620271	
		*Sturnira lilium*		ES	KF557744	KF557735	
*T. cruzi marinkellei*		*Phyllostomus discolor*		BA	AJ009150	AJ620270	
		*Phyllostomus* sp.		MA	KP197159	KP197169	
*T. cruzi*		*Phyllostomus hastatus*		MA	KP197160	KP197170	
		*Didelphis marsupialis*		AM	AF239981	GQ140351	
		*Homo sapiens*		SP	AF301912	GQ140353	
		*Homo sapiens*		AM	AY491761	GQ140355	
		*Panstrongylus geniculatus*		AM	AF288660	GQ140356	
		*Homo sapiens*		CH	AF228685	GQ140357	
		*Myotis levis*		SP	FJ001634	GQ140358	
*Phytomonas* sp.					AF016322	AF047496	

*Note*: Sequences generated in this study and deposited in GenBank are indicated in bold

*Abbreviations*: CBT, Coleção Brasileira de Tripanossomatídeos; ES, Espirito Santo; SP, São Paulo; BA, Bahia; MS, Mato Grosso do Sul; AM, Amazonas; MA, Maranhão; BR, Brazil; MY, Malaysia; CH, Chile; UK, United Kingdom

### Genomic DNA extraction and characterization of *T. terrestris* isolates

Genomic DNA from cultured trypanosomes was extracted from pellets of approximately 10^6^ parasites using the traditional phenol-chloroform method [33]. The DNA samples were subjected to the polymerase chain reaction (PCR) for three nuclear regions, including the *gGAPDH* gene, the V7V8 region of *SSU* rDNA and the ITS1 rDNA region, under amplification conditions that had previously been described [19, 34–36]. The amplicons were viewed on 1.5% agarose gels, which were stained with SYBR^®^ safe DNA gel stain (Thermo Fisher Scientific, Cambridge, MA, USA). The PCR products were purified by means of ExoSAP-It^®^ (Thermo Fisher Scientific) and were subjected to sequencing reactions using the BigDye^®^ Terminator cycle sequencing kit (Thermo Fisher Scientific). Automated sequencing was carried out in an ABI Prism 3500 genetic analyzer (Thermo Fisher Scientific) in accordance with the manufacturer’s instructions. Chromatograms were checked through SeqScape^®^ v.2.6.0 (Thermo Fisher Scientific). We phased *gGAPDH*, V7V8 *SSU* rDNA and ITS1 rDNA loci in DNAsp v.5.10.01 [37] using standard parameters, since several exploratory analyses yielded identical results.

The sequences obtained were used to generate multiple sequence alignments through Muscle v.3.8 [38], using standard settings, with manually adjustment through GeneDoc v.2.6.01 [39]. Based on previously available GenBank sequences for trypanosomatid species, additional alignments for phylogenetics and population genetic analyses were constructed. To add possible representative members of the *T. terrestris* clade and based on blast-n search, we added *SSU* rDNA and gGAPDH sequences (GenBank: GQ864158–GQ8641559) of a recently described trypanosome species (*Trypanosoma vanstrieni*) isolated from Southeast Asian rhinos (*Dicerorhinus sumatrensis*; Perissodactyla: Rhinocerotidae). New *T. terrestris* sequences retrieved from this study were deposited in the GenBank database (Table 1).

### Population genetic structure

General statistics for sequence diversity at each locus were calculated in DNAsp v.5.10.01, including the number of haplotypes (h), haplotype diversity (Hd), number of polymorphic sites (S) and nucleotide diversity (π). Because the ITS1 rDNA region is highly polymorphic and is widely used to assess intraspecific diversity in trypanosomes and pathogens [34, 36, 40], we constructed the ITS1 haplotype network using popART v.1.7 [41]. Based on ITS1 haplotypes (Additional file 2: Table S2) the degree of genetic isolation and population differentiation within and between parasite populations were estimated through F-statistics and absolute divergence measure (Nei’s Da) in Arlequin v.3.5.2.2 [42]. As we were interested in examining patterns of parasite genetic variation to test possible scenarios of host-switching and cospeciation, ITS1 haplotypes were grouped using two deme schemes: (i) according to patterns observed in haplotype networks; and (ii) according to geographical origin (biome) where parasites and hosts were collected (Table 1). The statistical significance of F_ST_ values was tested in analyses of molecular variance (AMOVA) based on three replicates of 20,000 random permutations per replicate, in Arlequin v.3.5.2.2. *P*-values were adjusted and corrected using the *SGoF* package [43] in R v.3.2.5 (R Development Core Team 2008). Fisher’s exact test of population differentiation based on haplotype frequencies with 100,000 iterations and 10,000 dememorization steps was also performed in Arlequin v.3.5.2.2.

In addition, by using all loci we determined the putative number of genetically distinct parasite population clusters (K) with the Bayesian program STRUCTURE v.2.3.4 [44]. This clustering method probabilistically assigns individuals (our individual parasite isolates) to populations based on sets of allele frequencies at each locus that are unique to each population. We evaluated admixture *vs* no-admixture (absence of gene flow) models, both assuming correlated allele frequencies. For each run, we used 50,000 MCMC (Markov Chain Monte Carlo) steps as ‛burn-inʼ followed by 100,000 steps for each K (1–8), with 10 replicates. STRUCTURE results were summarized in STRUCTURE HARVESTER [45], including identification of optimal K through the Evanno method [46]. The results from each replicate run were combined using CLUMPP [47] and viewed through DISTRUCT v.1.1 [48].

### Parasite population dynamics and isolation test

Examining the causes of genetic variation in parasite populations is often challenging and difficult to track. In this regard, some trypanosomes are known to exhibit complex life-cycles involving multiple hosts and/or vector species, and there are many cases where the vector species is not yet identified [19] as is the case of *T. terrestris* [29]. To overcome this, we observed patterns of genetic variation and parasite population dynamics based on a host capture–recapture strategy (tapir radio tagging over years 2012–2015) at microgeographical scale within the Nhecolândia landscape in Pantanal (14 km^2^), from which the samples were collected. First, given the positive hemocultures, we evaluated the parasite occurrence by observing the ratio between tapirs collected *vs* tapirs infected. Secondly, by using genetic data to access different parasite strains and haplotypes (Additional file 2: Table S2, Table 1) we searched for possible barriers to gene flow in *T. terrestris*, at three possible levels of genetic variation as follows: geographical, tapir hosts and ‘putative’ vectors. As we stated, if geography and tapir hosts are promoting the isolation of parasites within and among populations, the most plausible scenario is specialization/cospeciation. Alternatively, if genetic barriers are explained neither by geography nor by hosts, we must consider vectors as promoters of host-switching. Because the vector of *T. terrestris* is unknown and in the absence of a better definition, hereafter the term ‘putative vector’ can be equally regarded as ‘hypothetical’ or ‘possible vector’. To obtain a realistic assumption of possible genetic barriers within the Nhecolândia landscape, we used the software BARRIER v.2.2 [49]. This software implements Monmonier’s maximum difference algorithm to identify genetic boundaries between pairs of haplotypes, through data visualization on a map. Statistical support (robustness of putative barriers) was estimated by bootstrapping the genetic distance matrices of ITS1 rDNA haplotypes (100 replicates) using K2-P distances in the PHYLIP package v.3.6 [50] through DNADIST and SEQBOOT. We did not include the Atlantic forest samples (Table 1) for this analysis due to their lack of genetic variation and large geographical distance between biomes.

### Demographic history

We looked for genetic signatures of population contraction or expansions using all *T. terrestris* isolates (Table 1) through a coalescent extended Bayesian skyline plot (EBSP) [51]. This method uses multilocus data to plot effective population size through time, thereby providing a temporal reference of demographic events such as bottlenecks and expansions [51]. To provide such temporal reference, we configured the prior parameter belonging to mutation rates based on independent runs for each of the nuclear loci used. In this regard, we settled the tMRCA in our *T. terrestris* isolates pursuant to the origin of their lowland tapir hosts at approximately 3 Ma, as seen in previous molecular studies on this host [52] (see Additional file 3: Table S3). Considering that overall molecular clock hypotheses may be appropriate at the intraspecific level of molecular evolution, and because there was not much variation among *T. terrestris* intraspecific data used, we enforced a strict clock to simplify the model and help the analyses converge, as previously suggested [53].

To validate the EBSP, we conducted Tajima’s D and Fu and Li’s F neutrality tests in DNAsp, with our nuclear data. To prevent selection or genetic structure from affecting demographic trends, the analysis was performed with and without divergent isolates (CBTs 94, 97 and 98) (Additional file 4: Figure S1a) [54]. Moreover, to avoid possible effects of time constraints on our demographic analyses, we performed the same analysis in absence of time calibration points (Additional file 4: Figure S1b). Finally, we performed the EBSP by sampling from prior parameters in BEAST, to observe whether these drove the results.

### Phylogenetic relationships and divergence time estimation

Phylogenies including divergence times were estimated in BEAST v.2.2.1 [55] by using concatenated *gGAPDH* and V7V8 *SSU* rDNA gene alignments. The phylogenetic analysis included all isolates of *T. terrestris*, the trypanosome species retrieved from *D. sumatrensis* rhinos and members of *T. cruzi* and *T. grayi* clades (Table 1). To simplify the molecular clock assumption and thus circumvent possible bias in estimating divergence times, we did not add more trypanosome species due to great differences in molecular evolutionary rates of trypanosomes and because *T. cruzi* and *T. grayi* clades were previously placed as close relatives in phylogenomic studies [56]. Moreover, observations made by Acosta et al. [29] included high genetic similarity between *T. grayi* and *T. terrestris.*

Considering that estimation of absolute divergence times in trypanosomes is not straightforward because of the absence of fossil record [57, 58], to estimate the tMRCA in the *T. terrestris* clade we followed two calibration strategies. A first calibration point was placed at 479 Ma (normal distribution, σ 15.4 Ma) at the root of trypanosomes, based on the origin of insects according to insect phylogenomic studies [59], and assuming the ‘insect hypothesis’ as the most plausible one for the origin of trypanosomes [19]. A second calibration point was assumed at 60 Ma (normal distribution, σ 2.0 Ma), considering the origin of perissodactyl hosts, where trypanosomes from this study were isolated [60]. To set appropriate prior parameters in BEAST, we conducted several exploratory runs ranging from 10 to 20 × 10^6^ MCMC steps, sampling every 1000–2000 generations. A global strict clock was rejected, given that 95% HPD (highest posterior density) limits for coefficients of variation reached values close to one in all relaxed analyses. Thus, by employing a yule-tree prior parameter with an uncorrelated relaxed exponential clock for final runs, we ran two independent analyses of 70 × 10^6^ MCMC steps, sampling every 7000 generations. Models of DNA evolution were determined during each BEAST run through bModelTest [61], using empirical frequencies and transition/transversion split setting. We checked stationarity and convergence in the chains using Tracer v.1.6 [62], and the analyses were combined in LogCombiner v.2.2.1 [55] after discarding 20% as ‛burn-inʼ. We used TreeAnnotator v.2.2.1 to obtain the maximum clade credibility (MCC) tree [55], and FigTree v.1.4.2 [63] to edit and view the final tree.

We corroborated tree topologies and node statistical support by conducting an additional phylogenetic analysis using MrBayes v.3.1.2 [64] for 10^7^ MCMC generations and sampling parameters and trees every 2000 steps. After visual examination of log output in Tracer v.1.6, we discarded the first 25% of the trees as ‛burn-inʼ, and the remaining trees were used to calculate the Bayesian posterior probabilities.

## Results

### Population genetic structure

**Fig. 2:**
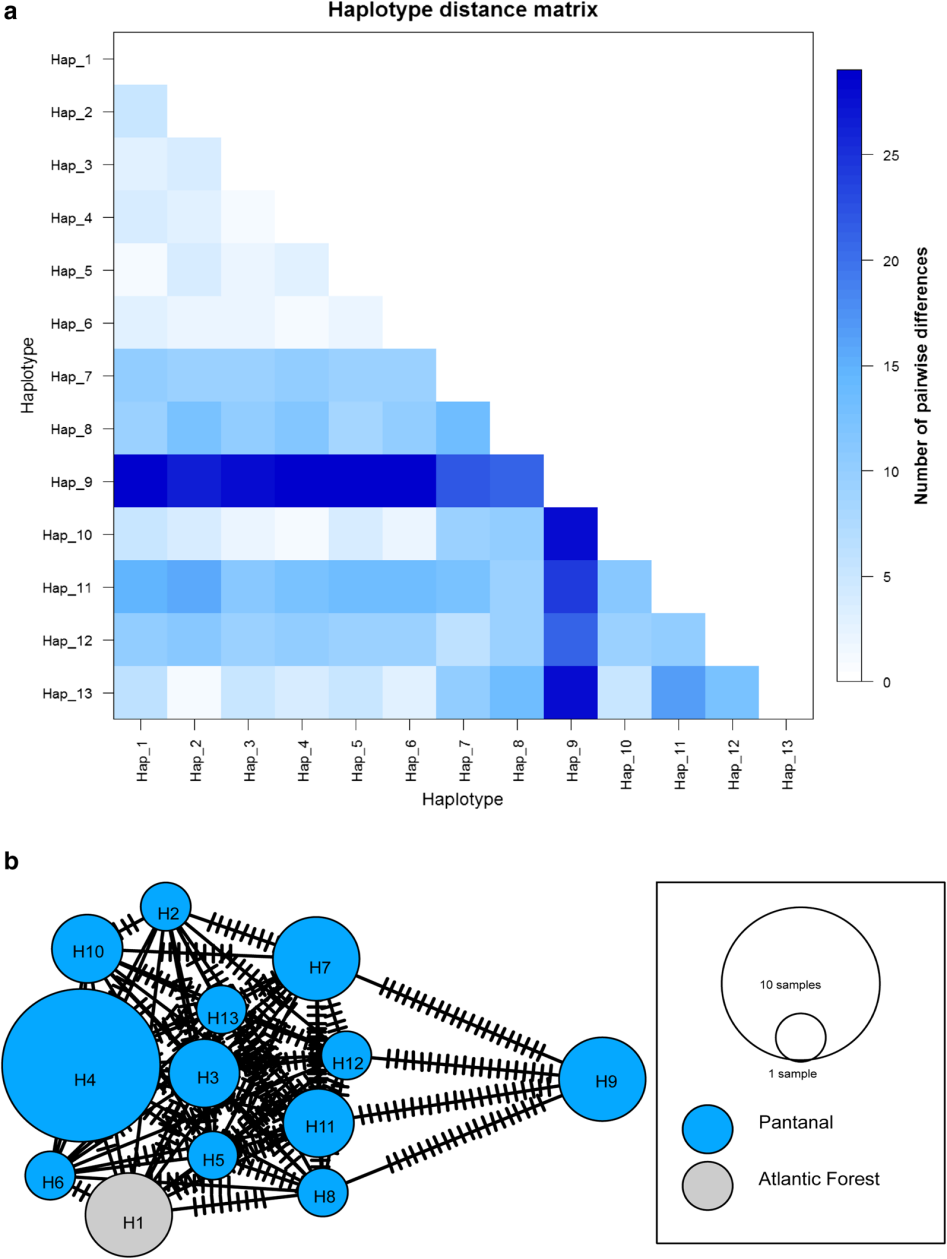
Patterns of genetic variation in *T. terrestris.* **a** Heat map of Nei’s genetic distances showing the average number of pairwise differences between ITS1 rDNA haplotypes. **b** ITS1 rDNA haplotype network inferred by minimum spanning network. Circle sizes correspond to the frequency of CBTs per haplotype and vertical lines connecting the network represent the number of mutations

Isolates of *T. terrestris* examined in this study were quite homogeneous and overall had low nucleotide genetic diversity among loci. ITS1 rDNA was the most polymorphic marker and thus the most suitable for inferring genetic structure (Table 2). In addition, as expected with generalist parasites, there was no evidence of genetic differentiation neither by geography nor by tapir hosts. In this regard, from the 28 isolates belonging to the Pantanal, 12 ITS1 haplotypes were recorded (*n* = 12), and considering the 3 isolates from Atlantic Forest, only one haplotype was recorded (*n* = 1) (Additional file 2: Table S2; Fig. 2a, b). Despite F_ST_ being moderately high, no significant difference in genetic structure was detected between these biomes (*F*_ST_ = 0.171, *P* > 0.05) and in agreement with its large geographical scale and ecological disparity between Atlantic Rainforest and Pantanal, most of the variation was detected within the isolates from the Pantanal biome (Table 3).

**Table 2:** Summary statistics and neutrality tests

	Diversity indices					Neutrality tests	
	*n*	S	h	Hd	π	Fu & Li^’^s F	Tajima’s D
ITS1 (266 bp)							
Pantanal + Atlantic Forest	31	32	13	0.877	0.0348	1.265 (*P *> 0.1)	0.00199 (*P *> 0.1)
*gGAPDH* (695 bp)							
Pantanal + Atlantic Forest	31	4	5	0.570	0.0009	− 1.111 (*P *> 0.1)	− 0.89400 (*P* > 0.1)
V7V8 *SSU* rDNA (728 bp)							
Pantanal + Atlantic Forest	31	5	3	0.529	0.0010	− 2.328 (*P* > 0.1)	− 1.08769 (*P* > 0.1)

*Notes*: The length for each DNA region is listed (base pairs, bp). Neutrality tests are considered statistically significant if *P* < 0.05

*Abbreviations*: *n*, number of sequences; S, number of polymorphic sites; h, number of haplotypes; Hd, haplotype diversity; π, nucleotide diversity

**Table 3:** AMOVA results

Grouping strategy	Source of variation	*df*	SS	% variation	*F*_ST_	*P*-value
Pantanal + Atlantic Forest	Between populations	1	9.923	17.14	0.171	> 0.05
	Within populations	29	135.659	82.86		
	Total	30				
(Pantanal + Atlantic Forest) + (H9)	Between populations	1	65.719	80.84	0.808	< 0.0001*
	Within populations	29	79.863	19.16		
	Total	30				

*Note*: α value for all permutations was 0.01

*Fisher’s exact test of population differentiation: *P* < 0.0001 (CI: 0.0087±0.00094); *P*-value remained significant after correction

*Abbreviations*: *F*_ST_, fixation index; *df*, degrees of freedom; SS, sum of squares

**Fig. 3:**
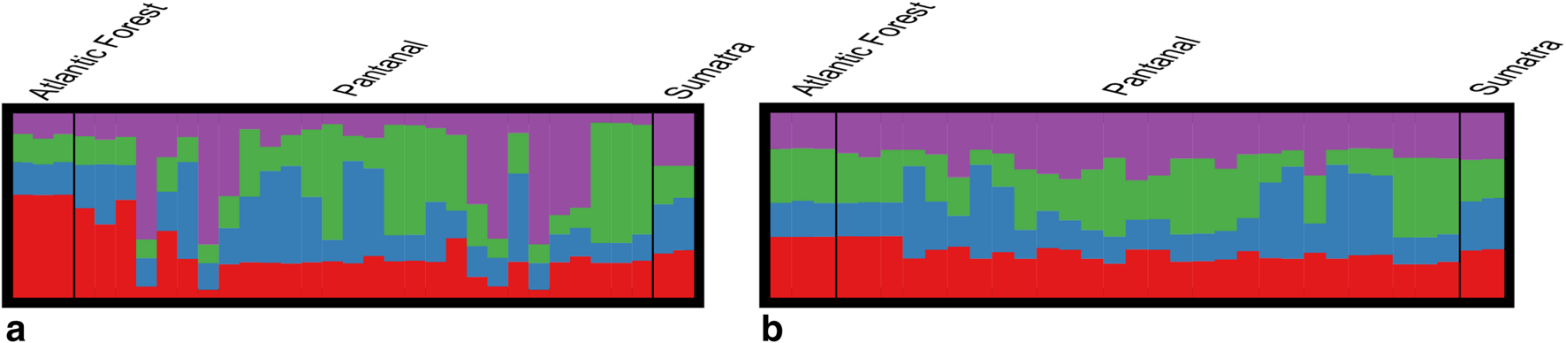
Results of the Bayesian clustering analysis in STRUCTURE. **a** Admixture model. **b** Non-admixture model. Isolates from Sumatra represent the trypanosomes of rhinos isolated from Southeast Asia (*Trypanosoma vanstrieni*)

Similarly, Bayesian clustering analysis in STRUCTURE showed no evidence of genetic structure, by which the *T. terrestris* isolates lacked enough genetic variation to be assigned into a particular genetic cluster (Fig. 3a, b). Although the optimal value of K populations was determined to be four (K = 4) (Additional file 5: Figure S2a, b), the software could not assign the cluster to which each isolate belonged, because in the face of the genetic homogeneity, at least 25% of the genotype isolates still belonged to each of the clusters (e.g. one color) (Fig. 3a, b). Even after including the trypanosome species from Southeast Asian rhinos (*T. vanstrieni*), the structure plot remained the same and individual parasitic isolates were generally equally admixed (Fig. 3a, b), implying little genetic variation in trypanosomes isolated from rhinos.

In spite of this lack of genetic structure, strikingly, haplotype networks and measures of genetic divergence detected a strongly divergent ITS1 haplotype at a microgeographical scale in the Nhecolândia landscape in the Pantanal: haplotype 9, H9 [(CBTs 94, 97 and 98) ~ 14 km^2^] (Fig. 2a, b). In that sense, there was an extraordinarily high degree of genetic differentiation between this divergent haplotype and the remaining haplotypes (*F*_ST_ = 0.808, *P* < 0.0001; Table 3), implying that other forces distinct from biome features or tapir hosts such as host-switching and/or eventual population bottlenecks are shaping this pattern of divergence. To avoid that this haplotype was not an artefact due to sequencing errors, we independently re-sequenced isolates 94, 97 and 98 and the haplotypes remained identical (Additional file 2: Table S2).

### Parasite population dynamics and isolation test

During the extensive fieldwork conducted over the years 2012–2015, we documented high levels of parasite occurrence, indicating that transmission and maintenance of the parasite *T. terrestris* to their tapir hosts is a persistent and continuous feature in space and time. Of a total of 24 surveyed tapirs, 6 tapirs were recaptures, and from 32 hemocultures, only 4 cultures were negative (28 positive), from which two tapirs identified with the labels ‛31 Jordanoʼ and ‛28 Dudaʼ were positive for trypanosome culture in the first capture, but negative in the second capture. Importantly, divergent isolates 94, 97 and 98 were recovered from tapirs frequently infected with other isolates (e.g. ‛15 Morenaʼ), also implying that sympatric tapir hosts are not barriers for these isolates (Table 1).

**Fig. 4:**
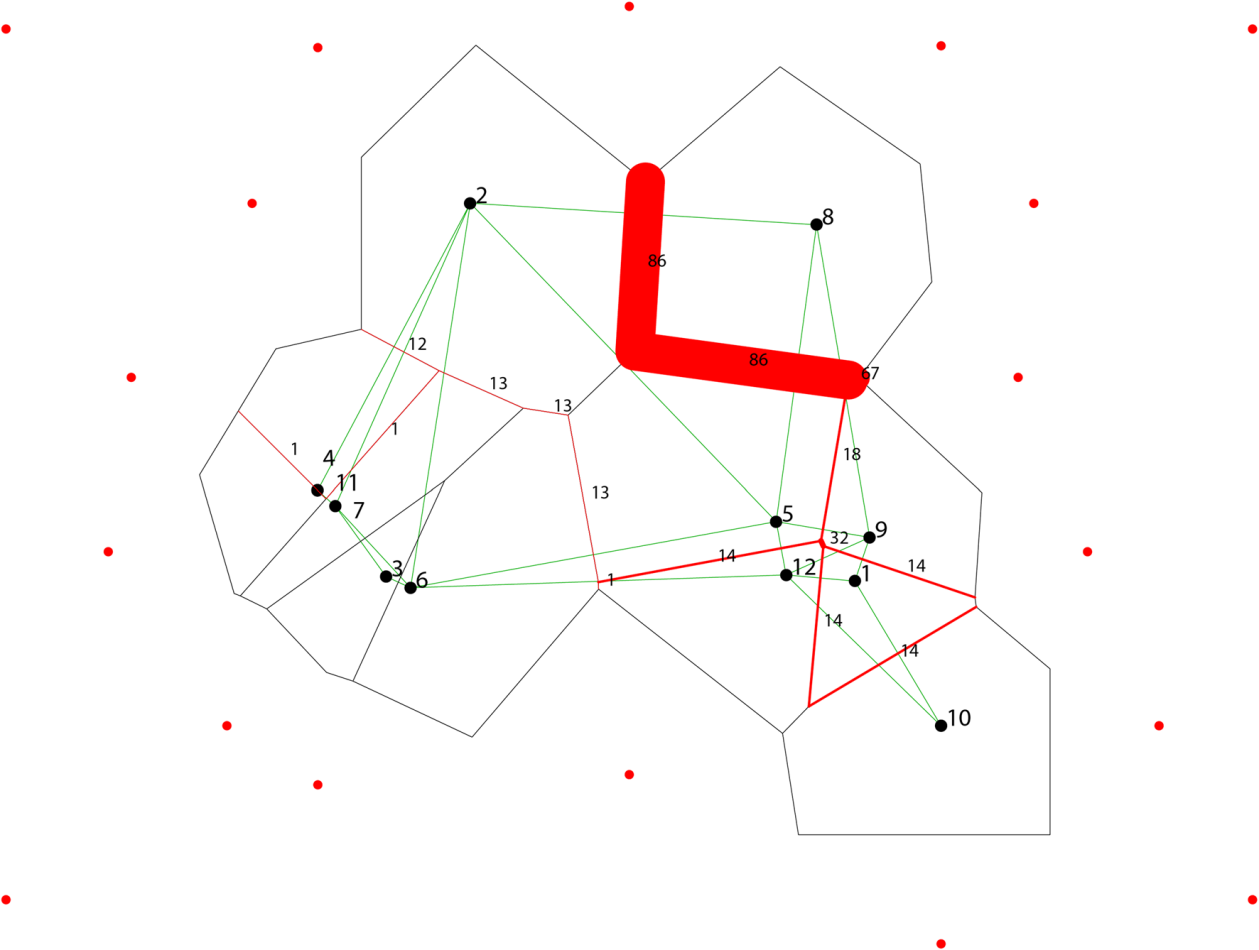
Results of the BARRIER test based on the bootstrapping of 100 K2P genetic distance matrices (Kimura 2-parameter) obtained from the random sampling of ITS1 haplotypes. Black and green lines represent the Voronoi/Delaunay tessellation/triangulation and the dots correspond to the geographical origin of ITS1 haplotypes sampled in Nhecolândia. Thickness of the red lines corresponds to the barrier robustness, identified by Monmonier’s maximum difference algorithm. In this case, sample 8 that has a bootstrap value of 86% is the genetic barrier belonging to H9

In the same way, results of the Barrier software yielded strong bootstrap support for the presence of a genetic barrier promoting the isolation of the haplotype 9 (H9) (Fig. 4). This result also corroborates that neither geography nor tapir hosts is causing this pattern, since Nhecolândia is a continuous floodplain, and tapirs carrying different parasite isolates were collected across the landscape (Fig. 4, Table 1), therefore suggesting that ‘putative’ vector species are promoting this divergence pattern through host-switching.

### Population bottleneck

**Fig. 5:**
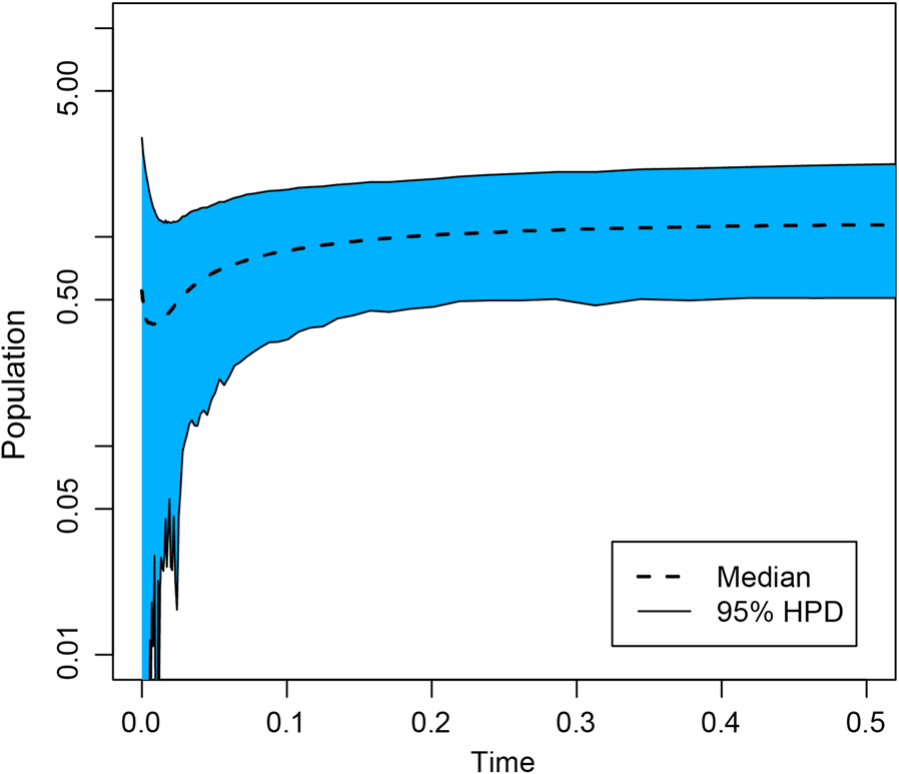
Extended Bayesian skyline plot illustrating the entire posterior distribution of demographic trends for *T. terrestris* isolates employed in this study. Dashed lines are the median effective population sizes, whereas the solid ones belong to 95% HPD limits. The time is in units of million years before present and population uses a logarithmic scale (Log 4Neµ)

In line with the general lack of genetic structure and low genetic diversity observed in the *T. terrestris* isolates employed (Tables 2, 3; Figs. 2, 3), the EBSP recovered a sharp reduction in parasite population sizes (Fig. 5). Following this population decline, no evidence of recovery or population expansion was detected. When adding calibration points based on the emergence of wild lowland tapir hosts (Additional file 3: Table S3), the parasite population bottleneck was estimated at 0.1 Ma, i.e. since the last glacial period in the late Pleistocene. However, we must acknowledge the large Bayesian confidence intervals in the demographic plot (Fig. 5), therefore we cannot explicitly attribute the parasite population decline to this climate event.

Sampling from prior parameters in BEAST did not affect the demographic trends (constant population sizes in all time reference points) and neutrality tests did not show any evidence of selection (*P* > 0.10; Table 2). When CBTs 94, 97 and 98 (H9) were removed from the analysis, the demographic decline remained the same, showing that the population bottleneck was not affected by genetic structure (Additional file 4: Figure S1a). Similarly, the population decline was the same in the absence of time calibration points (Additional file 4: Figure S1b).

### Reconstructing the evolutionary history of *T. terrestris*

**Fig. 6:**
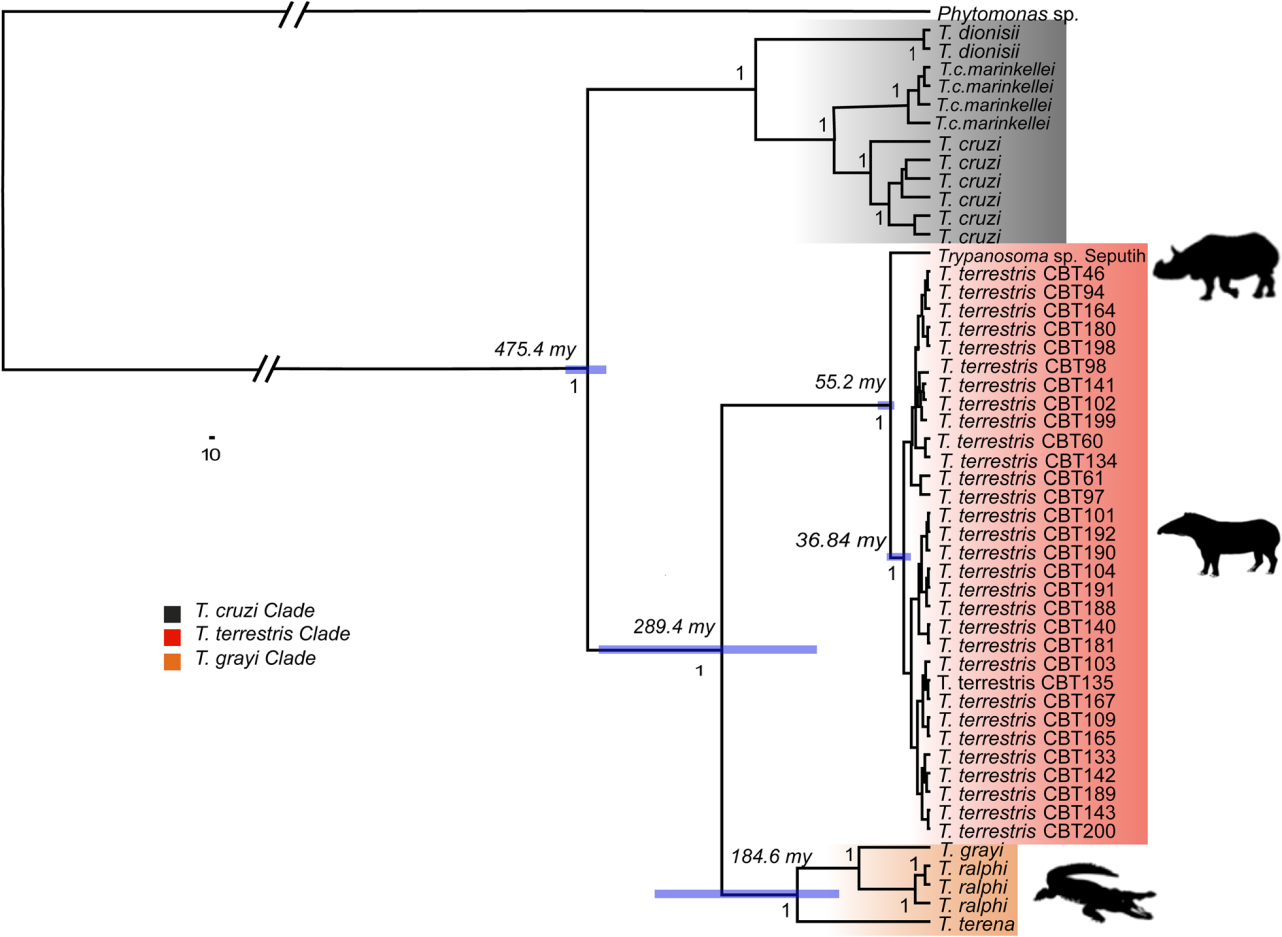
Maximum clade credibility tree (MCC) inferred by *gGAPDH* and V7V8 *SSU* rDNA concatenated sequences, showing phylogenetic relationships and divergence times (tMRCA) of studied trypanosomes. Posterior probability at each node is indicated by values ranging from 0.0 to 1.0. Horizontal blue bars represent the posterior credibility limits (HPD) for divergence time estimates

All *T. terrestris* isolates from Brazilian Pantanal and Atlantic Forest biomes retrieved from their lowland tapir host, *T. terrestris*, were grouped in a single and highly supported monophyletic clade with *T. vanstrieni*, isolated from Southeast Asian rhinos, *D. sumatrensis*, (posterior probability, pp = 1.0; Additional file 6: Figure S3; Fig. 6), suggesting that these trypanosome species are strongly specialized to their perissodactyl hosts. Furthermore, by means of a calibration point based on the estimated origin of Perissodactyla at 60 Ma (normal distribution, σ 2.0 Ma), the tMRCA in *T. terrestris* was estimated at ~ 37 Ma (95% HPD: 22.52 ± 49.6 Ma) (Fig. 6).

Nevertheless, although there is a close relationship between the *T. terrestris* clade and its mammal perissodactyl hosts (Fig. 6), *T. terrestris* shares a common ancestor with trypanosomes from reptiles, since it was placed as the sister clade of *T. grayi*, whose members include trypanosomes isolated from alligators of South America and crocodiles from Africa (Fig. 6) (posterior probability, pp = 1.0). Bayesian phylogeny inferred in MrBayes resulted in the same tree topology as the BEAST MCC tree, with equally robust posterior probabilities (Additional file 6: Figure S3).

## Discussion

Understanding the causes of genetic variation in parasites and how they are adapted to their hosts is a core question in evolutionary biology, with deep implications in the management and prediction of infectious disease dynamics [11, 12]. By using the recently described wild trypanosome species *T. terrestris* as a target organism, we herein present a double line of evidence suggesting that both host-switching by ecological fitting and coevolution are two important and non-mutually-exclusive processes driving the evolution of trypanosomes. In an attempt to cover for possible sources of genetic variation in *T. terrestris*, we made inferences on parasite genetic structure at three levels, covering geography, tapir hosts and ‘putative’ vectors. As seen in other parasite systems, our results indicate that trypanosomes indeed have complex life-cycles, and such life history traits as interaction with landscape features and host dispersal and transmission dynamics affecting their population genetic structure are important predictors in producing macroevolutionary patterns [10, 65].

As it is generally accepted, host dispersal abilities and host specificity (i.e. specialism) are key determinants of genetic structure in parasites [65]. *Tapirus terrestris* is a large solitary mammal species with the broadest geographical distribution of all Neotropical tapirs, and with a well-defined phylogeographic structure due to its limited social behavior and low vagility [66, 67]. This means that under a coevolving scenario, by which parasites are strongly specialized to their hosts, it is likely to expect high levels of genetic structure between parasite populations infecting different hosts from the Atlantic Forest and the Pantanal biomes. Considering only population structure, our findings refute this hypothesis. Most compatible with a model of host switching wherein parasites are generalists, we did not find evidence of genetic structure constrained by biomes or tapir hosts (Figs. 2, 3 and 4; Tables 2, 3). Similarly, the existence of a strongly divergent ITS1 haplotype at a microgeographical scale [(H9: CBTs 94, 97, 98) ~ 14 km^2^] which is explained neither by geography nor by sympatric tapir hosts (Tables 1, 3; Fig. 4), further suggests that putative vector species are simultaneously taking blood meals from other host sources across the landscape. In line with other studies on trypanosomes, these results are also indicative that ecological host-fitting appears to be the most parsimonious explanation to these patterns [23, 26, 27].

However, the most parsimonious explanation is not necessarily the only one. One important limitation of this study was the low number of loci and parasite isolates employed, and there are a few reasons for that. Due to their elusive and solitary behavior, lowland tapirs are extremely difficult to capture in nature and although hemoculturing increases the chances for parasite isolation, levels of parasitemia are frequently low in sylvatic mammals, thus making parasite detection difficult. Moreover, we were unable to amplify additional loci, because *T. terrestris* DNA did not amplify using the available *T. cruzi* primers [36]. We tried to overcome these operational difficulties by examining population dynamics in *T. terrestris* based on a lowland tapir capture-recapture strategy covering a period of three years (Additional file 1: Table S1, Fig. 4). We documented high levels of parasite occurrence, suggesting that transmission and maintenance of the parasite *T. terrestris* to their tapir hosts is a persistent and continuous feature in space and time. In particular, we also observed that different individual tapirs were able to carry different parasite isolates, including those isolates belonging to the divergent haplotype (H9), implying that this pattern of microgeographic divergence occurs through sympatric tapir hosts (Additional file 1: Table S1, Fig. 4).

More important is the genetic signature of a sharp reduction in parasite population sizes in the recent past (Fig. 5), which is the most likely reason for the lack of genetic structure and low genetic diversity observed (Figs. 2, 3, Table 3). Because of their fragmented nature, complex life-cycles and short generation times, parasites suffer dramatic variations in population sizes and frequently recurrent bottlenecks affecting their population genetics and evolution seems to be a universal characteristic governing parasite biology [9]. While it is true that the median line representing the population showed a decrease in the recent past, in this study we were unable to attribute the parasite bottleneck to a specific historical event due to large confidence intervals estimated (Fig. 5). Even so, following a population bottleneck the original gene pool in a population is heavily reduced and the remaining population faces higher levels of genetic drift [9], thereby changing its genetic structure (Figs. 2, 3 and 4). For this reason, and in the light of the absence of a reliable historical explanation to these demographic trends, with our data we cannot attribute the current genetic structure in *T. terrestris* to host-switching by itself. We also cannot discard that prior to the population bottleneck the genetic structure of the parasite *T. terrestris* was geographically structured according to their lowland tapir hosts, implying specialization. This is perhaps the reason why the software STRUCTURE was unable to assign to which K population belonged each parasite isolate and these were generally equally admixed (Fig. 3a, b). Whatever the scenario, our results suggest that this population bottleneck is affecting the *T. terrestris* populations and there at least two possible and non-mutually exclusive explanations to the current parasite genetic structure, as follows.

Under a host specialization scenario, a population bottleneck in *T. terrestris* is a direct consequence of a decrease in population sizes of their lowland tapir hosts. *Tapirus terrestris* is a species at risk of extinction, catalogued as vulnerable [68]. In fact, contemporary human actions including deforestation and cattle ranching are leading to most tapir populations to local extinction in both Pantanal and Atlantic Forest biomes [69, 70], thereby affecting the parasite population dynamics and genetic structure (Figs. 2, 3, 4 and 5). In specialist parasites of endangered hosts, in the face of population bottlenecks, a correlation between low genetic diversity and high parasite load has been observed because parasites tend to demonstrate high occurrence due to their inability to counter-adapt [9, 71]. This scenario could be plausible for *T. terrestris* because we recorded high parasite occurrence through the Nhecolândia landscape in Pantanal (Additional file 1: Table S1). Importantly, it seems that trypanosome species isolated from rhinos, which have the same pattern in the STRUCTURE plot (Fig. 3a, b), is affected by population bottlenecks because the Sumatran rhino (*D. sumatrensis*) is a critically endangered species [72]. Indeed, in line with these observations, *T. vanstrieni* isolates were retrieved from four dead *D. sumatrensis* specimens that had been kept in captivity, with high levels of parasitemia (McInnes, personal communication).

Another scenario for explaining the current parasite genetic structure implies host-switching. We suspect that stability of tapir population and modifications in land use in the Pantanal [70] favorably increase the range for putative vector species to take blood meals from other, unrelated vertebrate hosts, giving greater opportunities to trypanosomes to colonize and persist in new environments. As predicted by ecological fitting [5], while differentiating (e.g. H9; Figs. 2, 4), parasites are able to persist in novel hosts while still exploiting their ‘ancestral’ tapir hosts. Thus, further demographic fluctuations such as population bottlenecks (Fig. 5) could be explained by founder events. This has been documented previously when parasitic species are introduced into new ecosystems, and as a direct consequence of exploiting new hosts, parasites display very low genetic diversity with resulting population bottlenecks [73]. Similarly, experimental infections have revealed that in the course of a single blood meal, populations of *T. brucei* experience sharp and recurrent bottlenecks during migration of parasites from the midgut to the salivary glands of tsetse flies, followed by a recovery of founding populations when infective metacyclic parasites are exposed to mice, resulting in the establishment of novel genetic variants [32]. In addition, it is worth mentioning that although lowland tapirs are solitary mammals, some social behavior and substantial home range overlap between individual tapirs have been demonstrated [67, 70]. This explains why studied tapirs were able to carry different parasite strains across the landscape (Additional file 1: Table S1, Fig. 4). The landscape of Nhecolândia is formed by a complex mosaic of different kinds of habitats and is strongly dependent on seasonal flooding [74]. Notably, these hosts can also exploit different sources of habitats, and due to their capability to swim, not only are they involved in the dispersal of the parasite through the Pantanal (Table 1; Fig. 4), they also increase the likelihood of coming into contact with possible *T. terrestris* vectors which may include generalist blood-sucking invertebrates such as hematophagous arthropods and leeches.

The pattern of microgeographic divergence found in an ITS1 rDNA haplotype is considered a relevant finding in this study (Figs. 2, 4, Table 3). Given the population bottleneck and overall genetic homogeneity found in the parasite isolates, it was not possible to capture additional genetic variation. The success of a host-switching is a process that can take thousands of generations to achieve and implies a combination of many other ecological factors including host choice and different mechanisms of genetic isolation [75]. Because we used neutral loci (Table 2), it was not possible to ascribe this microgeographic divergence pattern to local adaptation. While ITS1 rDNA is a viable marker for revealing cryptic patterns of genetic structure in trypanosomes (e.g. [34, 36, 40]) we believe that a more rigorous sampling across the genome of *T. terrestris* is required. In combination with parasite surveys at local scales including an array of candidate vertebrate/invertebrate hosts, it will potentiate further hypothesis testing of more complex demographic scenarios, including genomic regions affected by natural selection.

Correspondingly, our phylogenetic results allow us to confirm the population patterns observed above. On the one hand, *T. terrestris* is strongly linked to the evolutionary history of its perissodactyl hosts because it shares a common ancestor with the trypanosome species isolated from *D. sumatrensis* (*Trypanosoma vanstrieni*, McInnes et al., unpublished data), suggesting a coevolving scenario between Perissodactyla and their trypanosomes (Fig. 6). Additionally, as envisaged by Acosta et al. [29], *T. terrestris* and *T. grayi* are sister clades (Fig. 6), further indicating that host-switching is a common feature promoting trypanosome evolution [28].

The order Perissodactyla is an ancient and diverse group of mammals that became widely distributed across several continents (Asia, Europe, North America and Central-South America) since it arose in the Eocene [60]. By using only parasitic genetic data and a calibration strategy based on the origin of Perissodactyla, both estimated tMRCA and Bayesian confidence intervals of *T. terrestris* isolates employed in this study largely coincide with the oldest fossil record of Tapiridae for at least 41 Ma, including the additional radiation of extant tapir species at ~ 30 Ma [~ 37 Ma (95% HPD: 22.52 ± 49.6 Ma)] [76] (Fig. 6). Under a coevolving scenario, this finding suggests that *T. terrestris* clade may have originated in Asia [52, 60]. For instance, similar to that which occurred in other parasites such as *Trichinella* species and their mammal hosts [77], episodes of biogeographical expansion of Perissodactyla through Europe and North America including dynamics of extinction of tapirs (taxon pulses) could explain the origin and speciation of trypanosome parasites isolated from *T. terrestris*. In this regard, it would be important to see if through biotic expansions of the genus *Tapirus*, its trypanosomes also underwent speciation processes. For that reason, additional surveys for the presence of trypanosomes from mammals of Central and South America (*T. bairdii*, *T. pinchaque*) and Southeast Asia (*T. indicus*) are necessary.

The close relationship between *T. terrestris* and *T. grayi* clades is not surprising (Fig. 6). The phylogenetic closeness between *T. grayi* and *T. terrestris* can be explained by the fact that trypanosomes have been able to colonize distantly related hosts throughout their phylogenetic diversification [25, 28]. Therefore, our data suggest that relationships between ‘crocodilian’ and ‘mammalian’ clades are not strictly defined by their vertebrate hosts (Fig. 6), whereby trypanosomes of most mammalian orders are not monophyletic [28].

Taken together, our results suggest that niche overlapping by sharing vectors is a conservative trait explaining the *T. terrestris* and *T. grayi* relationships (Fig. 6). Tapirs and caimans shared continuously overlapped niches, ranging among swamp forests, moist woodlands and wetlands in South America [66, 78], providing the ideal arena for host shifts. Notwithstanding, the vectors of *T. grayi* are tsetse flies, and the vectors of *T. ralphi* and *T. terena* infecting South American alligators are currently unknown, but it has been suggested that generalist leeches are their vectors [79]. Several ectoparasites have been reported infecting lowland tapirs and could function as potential vectors, including ticks, hippoboscid dipterans, phlebotomines, culicids and tabanid flies [29]. Trypanosomes such as *T. theileri*, *T. evansi* and *T. vivax*, which infect artiodactyl ungulates, are transmitted by several hematophagous dipterans [35]. The vectors of *T. terrestris* and *T. vanstrieni* remain unknown, and further research on potential vectors and their ecological relationships with vertebrate hosts will provide valuable information on natural history and evolution of host specificity in these trypanosomes.

## Conclusions

Despite the special focus on human pathogenic trypanosomes, little attention has been focused towards trypanosomes infecting wild hosts. By using the recently described trypanosome species *T. terrestris* as a target organism, we present herein a double line of evidence, micro- and macroevolutionary, suggesting that both host-switching by ecological fitting and coevolution are two important and non-mutually-exclusive processes driving the evolution of trypanosomes. To our knowledge, this is the first time such evidence has been presented. Hence, although a scenario of cospeciation for *T. terrestris* is plausible, specialization does not configure an evolutionary dead-end and frequently host-shifts are not precluded. In this regard, we point out additional lines of evidence suggesting that even in the light of specialization, ecological opportunities for host-switching in trypanosomes are given by the ability of vectors to infect a wide range of hosts, and specifically niche overlapping by sharing vectors could be a conservative trait explaining the current *T. terrestris* and *T. grayi* relationships. The population bottlenecks as well as vertebrate/invertebrate host life history traits affecting dispersal and transmission dynamics of trypanosomes are important in determining the parasite genetic structure. We are convinced that the study of population dynamics of the vast majority of trypanosomes infecting sylvatic hosts will enhance our knowledge of trypanosomes in a broader sense, including the evolutionary mechanisms depicted by human pathogens such as *T. cruzi* and *T. brucei.*

## Supplementary information

**Additional file 1: Table S1.** List of tapirs sampled, parasite isolates and their geographical origin.

**Additional file 2: Table S2.** ITS1 haplotypes with associated CBTs, only considering polymorphic sites.

**Additional file 3: Table S3.** Mutation rates used in the demographic analysis. Several independent runs of 20~40 × 10^6^ MCMC generations were realized for each nuclear region, and using bModelTest as strategy to find DNA substitution models in BEAST v.2.2.1. Convergence of each run was checked in Tracer v.1.6. The tMRCA in our *T. terrestris* isolates was assumed according to the emergence of *Tapirus terrestris* in South America [normal distribution (3 Ma, σ 0.2 Ma)]. 95% highest posterior density (HPD) is the analogue of confidence intervals in Bayesian statistics. Units are in substitutions/site/million years.

**Additional file 4: Figure S1. a** Extended Bayesian skyline plot illustrating the entire posterior distribution of demographic trends for *T. terrestris* isolates after removing CBTs 94-97-98 [= haplotype 9 (H9)]. Dotted lines indicate median effective population sizes, whereas the solid ones belong to 95% HPD limits. The time is in units of million years before present and population is at a logarithmic scale. **b** Extended Bayesian skyline plot illustrating the entire posterior distribution of demographic trends in the absence of time calibration points. X-axis indicates mutation rates (substitutions/site) and Y-axis corresponds to population size × mutation rate (log scale).

**Additional file 5: Figure S2.** Evanno plot derived from STRUCTURE HARVESTER depicting the most likely number of genetic clusters. **a** No-admixture model. **b** Admixture model.

**Additional file 6: Figure S3.** Bayesian phylogeny estimated in MrBayes v.3.1.2 by using V7V8 *SSU* rDNA sequences and depicting the evolutionary relationships of *T. terrestris* clade and its close relatives. Blue dashed lines represent the *T. terrestris/T. grayi* monophyletic clade. Numerical values represent the Bayesian posterior probabilities ranging from 0.0 to 1.0.

## Data Availability

Data supporting the conclusions of this article are included within the article and its additional files. The newly generated sequences were submitted to the GenBank database under the Accession numbers shown in Table 1.

## Abbreviations

CBT: Brazilian trypanosomatid collection
EBSP: extended Bayesian skyline plot
HPD: highest posterior density
Ma: millions of years before present
MCC: maximum clade credibility
pp: posterior probability
tMRCA: time of the most recent common ancestor
