# Supplementary material for: Phylogenetics, patterns of genetic variation and population dynamics of Trypanosoma terrestris support both coevolution and ecological host-fitting as processes driving trypanosome evolution
Source: Parasit Vectors. 2019 Oct 11;12:473. doi: 10.1186/s13071-019-3726-y (PMC6790053; doi:10.1186/s13071-019-3726-y)
Supplement: Supplementary file 1 — Additional file 1: Table S1. List of tapirs sampled, parasite isolates and their geographical origin. [file 13071_2019_3726_MOESM1_ESM.docx]

**Additional file 1: Table S1.** List of tapirs sampled, parasite isolates and their geographic origin.

| *Tapirus terrestris* | | |  | *Trypanosoma terrestris* | |  |  |  |
| --- | --- | --- | --- | --- | --- | --- | --- | --- |
| ID/Name (capture/recapture) | Capture date | Sex/Age | Infectivity | CBT | Geographic origin/Biome | | Coordinates  Latitude/Longitude | |
| 32 Paulinho | 24.09.2012 | ♂/Juvenile | Positive | 94 | Nhecolândia | P | -19.26476 | -55.7590207 |
| 15 Morena | 20.09.2012 | ♀/Subadult | Positive | 97 | Nhecolândia | P | -19.317085 | -55.7535439 |
| 31 Jordano | 20.09.2012 | ♂/Subadult | Positive | 98 | Nhecolândia | P | -19.299746 | -55.8188741 |
| 31 Jordano | 27.10.2014 | ♂/Subadult | **Negative** |  |  |  |  |  |
| 08 Benjamin Martlet | 18.11.2012 | ♂/Adult | Positive | 101 | Nhecolândia | P | -19.309075 | -55.7542613 |
| 20 Sy | 15.07.2014 | ♀/Adult | **Negative** |  |  |  |  |  |
| 34 Carijó | 21.11.2012 | ♂/Subadult | Positive | 102 | Nhecolândia | P | -19.262074 | -55.8020845 |
| 28 Duda | 15.11.2012 | ♀/Adult | Positive | 103 | Nhecolândia | P | -19.308302 | -55.7628135 |
| 28 Duda | 20.08.2014 | ♀/Adult | **Negative** |  |  |  |  |  |
| 33 Gabriela | 21.11.2012 | ♀/Juvenile | Positive | 104 | Nhecolândia | P | -19.309905 | -55.8095123 |
| 35 Guilherme | 08.12.2012 | ♂/Adult | Positive | 109 | Nhecolândia | P | -19.308506 | -55.8125679 |
| 21 Caio | 25.05.2013 | ♂/Subadult | Positive | 133 | Nhecolândia | P | -19.301669 | -55.7640435 |
| 29 Karin Schwartz | 29.05.2013 | ♀/Adult | Positive | 134 | Nhecolândia | P | -19.308506 | -55.8125679 |
| 37 Justine | 30.05.2013 | ♀/Subadult | Positive | 135 | Nhecolândia | P | -19.317085 | -55.7535439 |
| 38 Cassandra | 21.07.2013 | ♀/Adult | Positive | 140 | Nhecolândia | P | -19.297808 | -55.8210674 |
| 39 Manfredo | 25.07.2013 | ♂/Subadult | Positive | 141 | Nhecolândia | P | -19.297808 | -55.8210674 |
| 15 Morena | 28.07.2013 | ♀/Subadult | Positive | 142 | Nhecolândia | P | -19.317085 | -55.7535439 |
| 16 Dora | 28.07.2013 | ♀/Adult | Positive | 143 | Nhecolândia | P | -19.303632 | -55.7524182 |
| 19 Sérgião | 20.10.2013 | ♂/Adult | Positive | 164 | Nhecolândia | P | -19.282518 | -55.7567555 |
| 30 Nelsão Chester | 20.10.2013 | ♂/Adult | Positive | 165 | Nhecolândia | P | -19.303632 | -55.7524182 |
| 41 Tinoco | 18.12.2013 | ♂/Subadult | Positive | 167 | Nhecolândia | P | -19.319946 | -55.7703877 |
| 43 Jujuba | 05.05.2014 | ♀/Juvenile | **Negative** |  |  |  |  |  |
| 42 Renatinha | 04.05.2014 | ♀/Juvenile | Positive | 180 | Nhecolândia | P | -19.279424 | -55.7938988 |
| 38 Cassandra | 07.05.2014 | ♀/Adult | Positive | 181 | Nhecolândia | P | -19.297808 | -55.8210674 |
| 29 Karin Schwartz | 12.07.2014 | ♀/Adult | Positive | 188 | Nhecolândia | P | -19.297808 | -55.8210674 |
| 36 Michele | 03.07.2014 | ♀/Subadult | Positive | 189 | Nhecolândia | P | -19.301669 | -55.7640435 |
| 33 Gabriela | 06.07.2014 | ♀/Subadult | Positive | 190 | Nhecolândia | P | -19.297808 | -55.8210674 |
| 44 Gabrielzinho | 13.07.2014 | ♂/Juvenile | Positive | 191 | Nhecolândia | P | -19.299746 | -55.8188741 |
| 34 Carijó | 06.07.2014 | ♂/Adult | Positive | 192 | Nhecolândia | P | -19.265268 | -55.8081984 |
| 38 Cassandra | 18.10.2014 | ♀/Adult | Positive | 198 | Nhecolândia | P | -19.308506 | -55.8125679 |
| 23 Rick Barongi | 21.10.2014 | ♂/Adult | Positive | 199 | Nhecolândia | P | -19.281909 | -55.7913114 |
| 45 Dona Jô | 22.10.2014 | ♀/Adult | Positive | 200 | Nhecolândia | P | -19.308506 | -55.8125679 |
|  |  |  |  |  |  |  |  |  |
|  |  |  |  |  |  |  |  |  |
|  |  |  |  | 46 | Linhares | AF | -19.083333 | -39.96667 |
|  |  |  |  | 60 | Pinheiros | AF | -18.3425 | -40.1466675 |
|  |  |  |  | 61 | Marechal Flor. | AF | -20.412778 | -40.683056 |

Shaded cells represent recaptured tapirs. CBT: Coleção Brasileira de Tripanossomatídeos/parasite isolate. P: Pantanal, AF: Atlantic Forest
