# Supplementary material for: Phylogenetics, patterns of genetic variation and population dynamics of Trypanosoma terrestris support both coevolution and ecological host-fitting as processes driving trypanosome evolution
Source: Parasit Vectors. 2019 Oct 11;12:473. doi: 10.1186/s13071-019-3726-y (PMC6790053; doi:10.1186/s13071-019-3726-y)
Supplement: Supplementary file 2 — Additional file 2: Table S2. ITS1 haplotypes with associated CBTs, only considering polymorphic sites. [file 13071_2019_3726_MOESM2_ESM.docx]

**Additional file 2: Table S2.** ITS1 haplotypes with associated CBTs, only considering polymorphic sites

|  |  | **Number of polymorphic sites** | | | | | | | | | | |  |  |  |  |  |  |  |  |  |  |  |  |  |  |  |  |  |  |  |  |  |
| --- | --- | --- | --- | --- | --- | --- | --- | --- | --- | --- | --- | --- | --- | --- | --- | --- | --- | --- | --- | --- | --- | --- | --- | --- | --- | --- | --- | --- | --- | --- | --- | --- | --- |
| **Hap** | **CBT** | 1 | 2 | 3 | 4 | 5 | 6 | 7 | 8 | 9 | 10 | 11 | 12 | 13 | 14 | 15 | 16 | 17 | 18 | 19 | 20 | 21 | 22 | 23 | 24 | 25 | 26 | 27 | 28 | 29 | 30 | 31 | 32 |
| **H1** | 46 60 61 | G | C | G | G | G | A | A | A | A | A | A | A | C | G | A | C | C | T | C | A | G | T | T | A | T | C | G | G | A | A | T | A |
| **H2** | 101 | . | G | . | . | . | G | . | C | . | . | . | . | . | . | . | . | . | . | . | . | . | . | . | . | . | . | . | . | . | T | . | T |
| **H3** | 102 192 | . | G | . | . | . | . | . | . | . | . | . | . | . | . | . | . | . | . | . | . | . | . | . | . | . | . | . | . | . | T | A | . |
| **H4** | ^a^ | . | G | . | . | . | . | . | . | . | . | . | . | . | . | . | . | . | . | . | . | . | . | . | . | . | . | . | . | . | T | A | T |
| **H5** | 141 | . | G | . | . | . | . | . | . | . | . | . | . | . | . | . | . | . | . | . | . | . | . | . | . | . | . | . | . | . | . | . | . |
| **H6** | 189 | . | G | . | . | . | . | . | . | . | . | . | . | . | . | . | . | . | . | . | . | . | . | . | . | . | . | . | . | . | T | . | T |
| **H7** | 190 104 109 | . | G | . | . | . | G | . | . | . | . | . | C | . | C | . | . | . | C | G | T | . | . | A | . | C | . | . | . | . | T | . | . |
| **H8** | 191 | . | G | . | . | . | . | . | . | . | . | C | C | . | C | C | . | . | . | . | . | A | . | . | C | . | G | T | . | . | . | . | . |
| **H9** | 94 97 98 | A | A | . | A | A | G | C | C | C | C | C | C | A | C | C | . | G | . | A | T | A | A | A | T | C | G | T | A | . | T | C | . |
| **H10** | 142 143 | . | G | . | . | . | . | . | . | . | . | . | . | . | C | . | . | . | . | . | . | . | . | . | . | . | . | . | . | . | T | A | T |
| **H11** | 134 135 | . | G | . | . | . | . | . | . | . | . | C | C | . | C | C | T | A | C | . | . | . | . | . | C | C | G | . | . | T | T | A | . |
| **H12** | 188 | . | G | . | . | . | . | . | . | . | . | C | C | A | C | . | . | . | C | . | . | A | . | A | . | C | . | . | . | . | T | . | . |
| **H13** | 103 | . | G | T | . | . | G | . | C | . | . | . | . | . | . | . | . | . | . | . | . | . | . | . | . | . | . | . | . | . | T | . | T |

^a^ CBTs of H4: 133 167 181 140 198 164 199 165 180 200
