# Supplementary material for: Phylogenetics, patterns of genetic variation and population dynamics of Trypanosoma terrestris support both coevolution and ecological host-fitting as processes driving trypanosome evolution
Source: Parasit Vectors. 2019 Oct 11;12:473. doi: 10.1186/s13071-019-3726-y (PMC6790053; doi:10.1186/s13071-019-3726-y)
Supplement: Supplementary file 3 — Additional file 3: Table S3. Mutation rates used in the demographic analysis. Several independent runs of 20~40 × 106 MCMC generations were realized for each nuclear region, and using bModelTest as strategy to find DNA substitution models in BEAST v.2.2.1. Convergence of each run was checked in Tracer v.1.6. The tMRCA in our T. terrestris isolates was assumed according to the emergence of Tapirus terrestris in South America [normal distribution (3 Ma, σ 0.2 Ma)]. 95% highest posterior density (HPD) is the analogue of confidence intervals in Bayesian statistics. Units are in substitutions/site/million years. [file 13071_2019_3726_MOESM3_ESM.docx]

**Additional file 3: Table S3.** Mutation rates used in demographic analysis. Several independent runs of 20~40x10^6^ MCMC generations were realized for each nuclear region, and using bModelTest as strategy to find DNA substitution models in BEAST v2.2.1. Convergence of each run was checked in Tracer v1.6. The tMRCA in our *T. terrestris* isolates was assumed according to the emergence of *Tapirus terrestris* in South America [Normal distribution (3 Ma, σ 0.2 Ma)]. 95% Highest Posterior Density –HPD- is the analogue of confidence intervals in bayesian statistics. Units are in substitutions/site/million year.

| **Region** | **Mutation rate**  **(sub/site/my)** | **95% HPD (sub/site/my)** |
| --- | --- | --- |
| **ITS1** | 1.74 x10^-2^ | 2.65 x10^-2^ ± 9.30 x10^-3^ |
| **gGAPDH** | 3.425x10^-4^ | 5.239 x10^-4^ ± 5.62 x10^-5^ |
| **V7V8 18S rDNA** | 2.66 x10^-4^ | 6.55 x10^-4^ ± 7.968 x10^-5^ |
