# Supplementary material for: Phylogenetics, patterns of genetic variation and population dynamics of Trypanosoma terrestris support both coevolution and ecological host-fitting as processes driving trypanosome evolution
Source: Parasit Vectors. 2019 Oct 11;12:473. doi: 10.1186/s13071-019-3726-y (PMC6790053; doi:10.1186/s13071-019-3726-y)
Supplement: Supplementary file 4 — Additional file 4: Figure S1. a Extended Bayesian skyline plot illustrating the entire posterior distribution of demographic trends for T. terrestris isolates after removing CBTs 94-97-98 [= haplotype 9 (H9)]. Dotted lines indicate median effective population sizes, whereas the solid ones belong to 95% HPD limits. The time is in units of million years before present and population is at a logarithmic scale. b Extended Bayesian skyline plot illustrating the entire posterior distribution of demographic trends in the absence of time calibration points. X-axis indicates mutation rates (substitutions/site) and Y-axis corresponds to population size × mutation rate (log scale). [file 13071_2019_3726_MOESM4_ESM.docx]

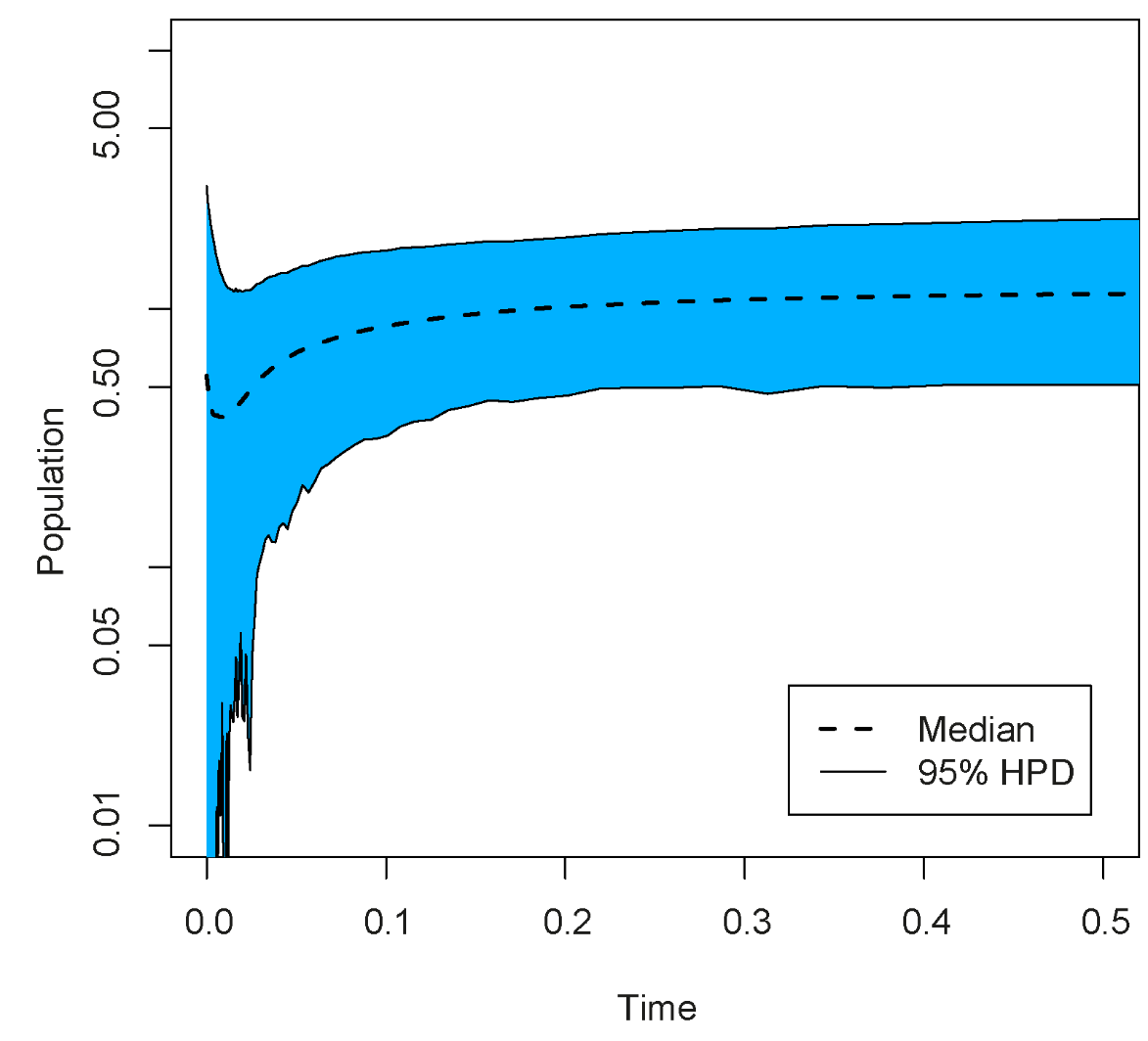


**Additional file 4: Figure S1a.** Extended Bayesian Skyline plot illustrating the entire posterior distribution of demographic trends for *T. terrestris* isolates after removing CBTs 94-97-98 (=Haplotype 9 –H9-). Dotted lines indicate median effective population sizes, whereas the solid ones belong to 95% HPD limits. The time is in units of million years before present and population is at logarithmic scale.


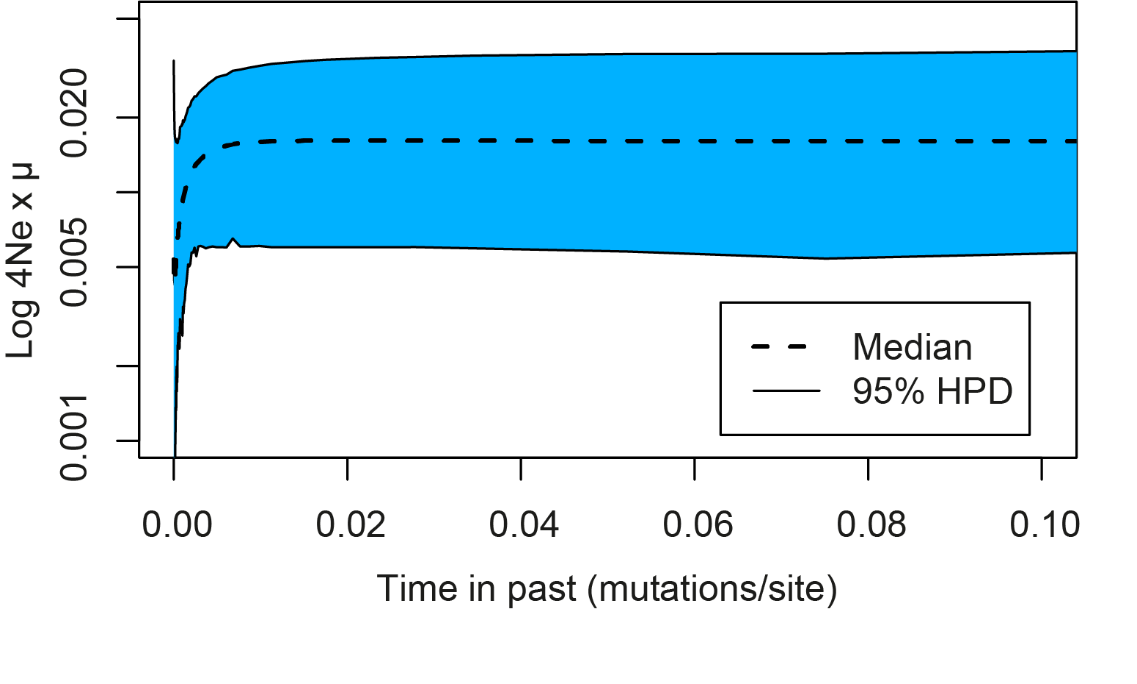


**Additional file 4: Figure S1b.** Extended Bayesian Skyline plot illustrating the entire posterior distribution of demographic trends in absence of time calibration points. ‘x’ axis is in mutation rates (substitutions/site) and ‘y’ axis corresponds to population size*mutation rate (log scale).
