# Supplementary material for: Phylogenetics, patterns of genetic variation and population dynamics of Trypanosoma terrestris support both coevolution and ecological host-fitting as processes driving trypanosome evolution
Source: Parasit Vectors. 2019 Oct 11;12:473. doi: 10.1186/s13071-019-3726-y (PMC6790053; doi:10.1186/s13071-019-3726-y)
Supplement: Supplementary file 5 — Additional file 5: Figure S2. Evanno plot derived from STRUCTURE HARVESTER depicting the most likely number of genetic clusters. a No-admixture model. b Admixture model. [file 13071_2019_3726_MOESM5_ESM.docx]

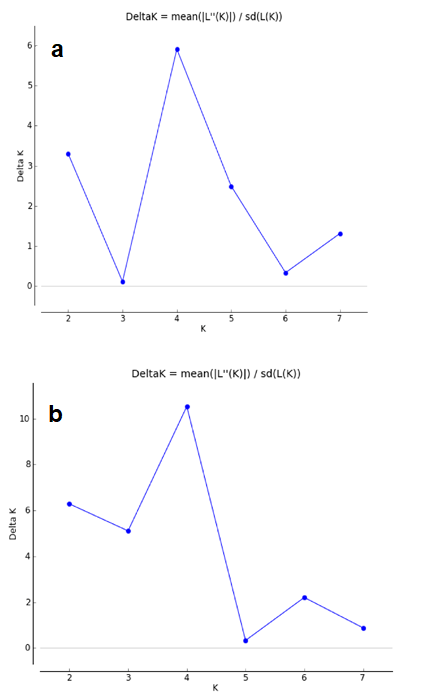


**Additional file 5:** **Figure S2.** Evanno plot derived from STRUCTURE HARVESTER depicting the most likely number of genetic clusters. **a**. No-admixture model. **b.** Admixture model.
