# Supplementary figures and images for: Phylogenetics, patterns of genetic variation and population dynamics of Trypanosoma terrestris support both coevolution and ecological host-fitting as processes driving trypanosome evolution
Source: Parasit Vectors. 2019 Oct 11;12:473. doi: 10.1186/s13071-019-3726-y (PMC6790053; doi:10.1186/s13071-019-3726-y)

Tree scale: 0.1

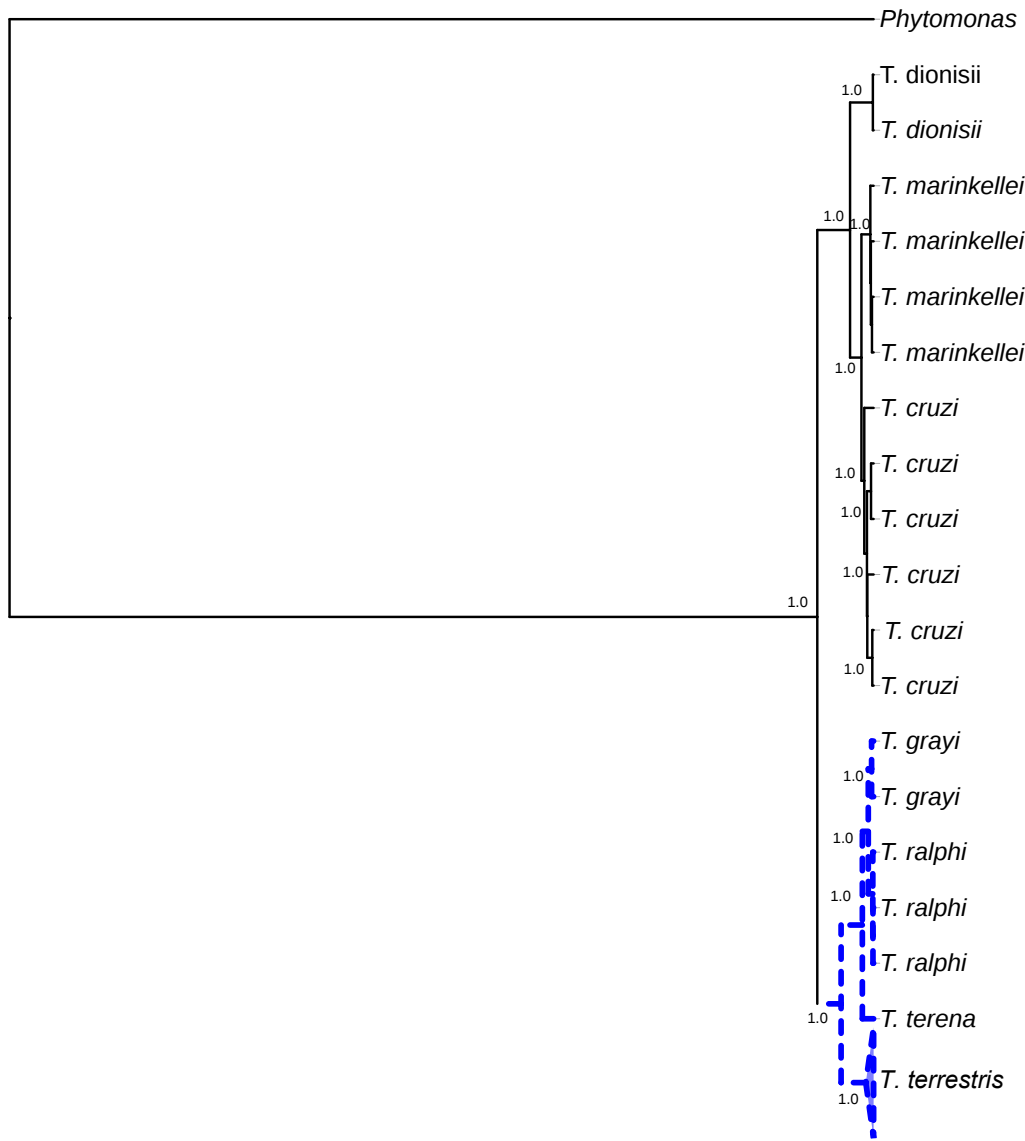

Supplement: Supplementary file 6 — Additional file 6: Figure S3. Bayesian phylogeny estimated in MrBayes v.3.1.2 by using V7V8 SSU rDNA sequences and depicting the evolutionary relationships of T. terrestris clade and its close relatives. Blue dashed lines represent the T. terrestris/T. grayi monophyletic clade. Numerical values represent the Bayesian posterior probabilities ranging from 0.0 to 1.0. [file 13071_2019_3726_MOESM6_ESM.pdf]
